# Dose-Additive Carcinogenicity of a Defined Mixture of “Dioxin-like Compounds”

**DOI:** 10.1289/ehp.7351

**Published:** 2004-10-19

**Authors:** Nigel J. Walker, Patrick W. Crockett, Abraham Nyska, Amy E. Brix, Michael P. Jokinen, Donald M. Sells, James R. Hailey, Micheal Easterling, Joseph K. Haseman, Ming Yin, Michael E. Wyde, John R. Bucher, Christopher J. Portier

**Affiliations:** ^1^National Institute of Environmental Health Sciences, National Institutes of Health, Department of Health and Human Services, Research Triangle Park, North Carolina, USA; ^2^Constella Group, Research Triangle Park, North Carolina, USA; ^3^Pathology Associates—A Charles River Company, Durham, North Carolina, USA; ^4^Experimental Pathology Laboratories, Research Triangle Park, North Carolina, USA; ^5^Battelle Columbus Laboratories, Columbus, Ohio, USA

**Keywords:** carcinogenicity, dioxin, mixtures, PCBs, persistent organochlorine pollutants, polychlorinated biphenyls, POPs, risk assessment, TEF, toxic equivalency factor

## Abstract

Use of the dioxin toxic equivalency factor (TEF) approach in human risk assessments assumes that the combined effects of dioxin-like compounds in a mixture can be predicted based on a potency-adjusted dose-additive combination of constituents of the mixture. In this study, we evaluated the TEF approach in experimental 2-year rodent cancer bioassays with female Harlan Sprague-Dawley rats receiving 2,3,7,8-tetrachlorodibenzo-*p*-dioxin (TCDD), 3,3′,4,4′,5-pentachlorobiphenyl (PCB-126), 2,3,4,7,8-pentachlorodibenzofuran (PeCDF), or a mixture of the three compounds. Statistically based dose–response modeling indicated that the shape of the dose–response curves for hepatic, lung, and oral mucosal neoplasms was the same in studies of the three individual chemicals and the mixture. In addition, the dose response for the mixture could be predicted from a combination of the potency-adjusted doses of the individual compounds. Finally, we showed that use of the current World Health Organization dioxin TEF values adequately predicted the increased incidence of liver tumors (hepatocellular adenoma and cholangiocarcinoma) induced by exposure to the mixture. These data support the use of the TEF approach for dioxin cancer risk assessments.

The human health risk posed by exposure to persistent organochlorine pollutants, including polychlorinated dioxins, polychlorinated dibenzofurans, and polychlorinated biphenyls (PCBs), present in the food and the environment is one of widespread concerns throughout the industrialized world (Hites et al. 2004; Stellman et al. 2003). Given the absence of adequate toxicology and carcinogenesis information on the vast majority of these classes, the dioxin toxic equivalency factor (TEF) approach is currently used worldwide for assessing and managing the risks posed by exposure to mixtures of certain dioxin-like compounds (DLCs) (Ahlborg et al. 1992; Birnbaum and DeVito 1995; Safe 1990; Van den Berg et al. 1998). The TEF approach is a relative potency paradigm that is based on estimates of the potency of dioxin-like effects of individual chemicals, or a mixture of chemicals assuming a common mechanism of action involving binding of the compound(s) to the aryl hydrocarbon receptor (AhR) (Schmidt and Bradfield 1996). Moreover, the risk associated with a mixture of DLCs may be estimated based on the effects of 2,3,7,8-tetrachlorodibenzo-*p*-dioxin (TCDD), the most potent member of this class of compounds, and using a dose metric that is based on the summation of the mass of each compound in the mixture after adjustment for its potency relative to that of TCDD. This concept of potency-adjusted dose additivity has been evaluated for a number of end points (DeVito et al. 1997; Hamm et al. 2003; Toyoshiba et al. 2004) but has never been evaluated for cancer risk from chronic/lifetime exposure. Given that assessments of human cancer risk are based in part on data obtained from rodent carcinogenicity studies, it is important and appropriate to assess whether the concept of dose additivity is valid for the carcinogenicity of a mixture of DLCs within the context of a rodent cancer bioassay.

To evaluate the TEF approach for the prediction of cancer risk, the National Toxicology Program (NTP) conducted multiple 2-year lifetime rat bioassays to evaluate the chronic toxicity and carcinogenicity of DLCs and structurally related PCBs and mixtures of these compounds. Specifically we conducted four 2-year rodent bioassays to test the hypothesis of dose-additive carcinogenicity of a defined mixture of DLCs. These studies were conducted in female Harlan Sprague-Dawley rats, based on the prior observations of the carcinogenic sensitivity to TCDD in the Spartan Sprague-Dawley rat strain (Kociba et al. 1978). Animals received either TCDD, PCB-126, 2,3,4,7,8-pentachlorodibenzofuran (PeCDF), or a mixture of all three compounds. Doses were established using the current World Health Organization (WHO) TEF values (Van den Berg et al. 1998) to provide doses of the individual chemicals or the mixture estimated to be equivalent to those used in the TCDD study. The mixture study was designed such that each compound would provide a third of the total dioxin toxic equivalents (TEQs) to the mixture. These relative levels were chosen to maximize statistical power to test for interactions between the three compounds. This does not reflect the relative abundance of each of these compounds in food, the primary medium of human exposure. However, these three compounds combined do account for approximately half of the dioxin-like activity found in human tissues. In this article, we report on the comparative dose–response modeling of the increases in incidence of specific neoplasms in these studies to test the hypothesis of dose additivity of carcinogenicity of dioxins within a defined mixture.

## Materials and Methods

### Animal use.

The animal studies were conducted at Battelle Columbus Laboratories (Columbus, OH). All studies were conducted according to Good Laboratory Practices (Food and Drug Administration 2002). Animals were obtained from Harlan SD (Indianapolis, IN) and upon receipt were approximately 6 weeks of age. They were held under quarantine for approximately 2 weeks for health screening and were approximately 8 weeks of age at the start of the study. After quarantine, the animals were randomly assigned to control or treated groups and permanently identified by tail tattoo. They were housed five per cage in solid-bottom polycarbonate cages (Lab Products, Inc., Maywood, NJ) suspended on stainless steel racks. Filtered room air underwent at least 10 changes/hr. Animal rooms were maintained at 69–75°F with 35–65% relative humidity and 12 hr light/12 hr dark. Irradiated NTP-2000 pelleted feed (Zeigler Bros., Inc., Gardners, PA) and water were available *ad libitum*. All animals were observed twice daily for morbidity checks and once per month for formal clinical signs of toxicity; moribund animals were euthanized and necropsied. The health status of the animals was monitored by serologic analysis of serum samples collected from the study animals and male sentinel rats that were placed in the study rooms. Serum samples remained negative for any significant rodent pathogen. Animal husbandry and handling were conducted in accordance with the National Institutes of Health guidelines (Institute of Laboratory Animal Resources 1996).

### Chemicals.

TCDD (lot no. CR82-2-2) was supplied by IIT Research Institute (Chicago, IL) and 3,3′,4,4′,5-pentachlorobiphenyl (PCB-126; lot no. 130494) by AccuStandard, Inc. (New Haven, CT). PeCDF (lot no. 080196) was purchased from Cambridge Isotope Laboratories (Cambridge, MA). Each chemical was received in one lot that was used for the entire study. Purity was determined several times during the study by gas chromatography/mass spectroscopy (GC/MS), nuclear magnetic resonance spectroscopy, and gas chromatography using flame ionization detection (PCB-126), electron capture detection (TCDD), proton and carbon-13 nuclear magnetic spectroscopy (PeCDF), and GC/MS (TEF mixture). Purities of TCDD, PCB-126, and PeCDF were determined to be approximately 98, 99.51, and 97%, respectively, with no change in purity observed over the duration of the studies. Dose formulations were prepared monthly for gavage administration by mixing the test chemical in a corn oil vehicle containing 1% USP-grade acetone. The corn oil was analyzed by potentiometric titration, and the acetone by infrared spectroscopy. Homogeneity and stability studies of dose formulations indicated that chemicals could maintain an acceptable homogeneity for dosing and stability for 35 days when stored at room temperature. Dose formulations were analyzed at least every 3 months and were within 10% of the target concentrations. For the mixture, the dose formulations were prepared by mixing volumes of the TCDD, PeCDF, and PCB-126 formulations.

### Treatment.

Animals were treated by gavage (2.5 mL/kg), 5 days per week for up to 2 years. Compounds used were TCDD, PCB-126, PeCDF, or a mixture of these three compounds. Group sizes for the 2-year carcinogenicity portion of these studies were 53 animals per dose group except for the 3-ng TCDD/kg group (*n* = 54) and the 30 ng PCB-126/kg group (*n* = 55). Target doses used for the individual compound studies were 3, 10, 22, 46, and 100 ng/kg TCDD; 30, 100, 175, 300, 550, and 1,000 ng/kg PCB-126; and 6, 20, 44, 92, and 200 ng/kg PeCDF. The TEF mixture was composed of equal ratios (1:1:1) of TEQs for TCDD, PCB-126, and PeCDF. The TEQ, calculated by multiplying the TEF value (Van den Berg et al. 1998) of each specific compound by the concentration of that compound in the mixture, results in the TCDD equivalent of that compound. For the TEF mixture, doses were formulated for comparison with the 10, 22, 46, and 100 ng/kg TCDD group by using the WHO TEFs of 1.0 for TCDD, 0.1 for PCB-126, and 0.5 for PeCDF. Specific target doses used in the TEF mixture study were 10 ng TEQ/kg (3.3 ng/kg TCDD, 6.6 ng/kg PeCDF, 33.3 ng/kg PCB-126), 22 ng TEQ/kg (7.3 ng/kg TCDD, 14.5 ng/kg PeCDF, 73.3 ng/kg PCB-126), 46 ng TEQ/kg (15.2 ng/kg TCDD, 30.4 ng/kg PeCDF, 153 ng/kg PCB-126), and 100 ng TEQ/kg (33 ng/kg TCDD, 66 ng/kg PeCDF, 333 ng/kg PCB-126). Control animals received corn oil:acetone vehicle (2.5 mL/kg) alone. Batches of actual dosing formulations used were periodically sampled and analyzed every 2–3 months by GC/MS to ensure that they were within 10% of the target concentration.

### Pathology.

At necropsy, all tissues were examined grossly, any lesions observed were recorded, and a full complement of tissues was removed and fixed in 10% neutral buffered formalin for microscopic evaluation. After fixation, the tissues were trimmed, processed, embedded in paraffin, sectioned at a thickness of 5 μm, stained with hematoxylin and eosin, and examined microscopically. The pathology findings from all studies were subjected to a full pathology peer review. To ensure consistency of the histopathologic diagnoses among the TEF dioxin projects, the same study pathologist, quality assurance pathologist, pathology working group (PWG) chairperson, NTP pathologist, and members of the PWG served in all studies. In addition, diagnostic criteria for the proliferative hepatocellular lesions were peer reviewed by an external expert panel advisory board.

### Statistical analysis.

Dose-specific tumor incidence was survival adjusted using the poly-3 adjustment (Bailer and Portier 1988). Data were modeled using a Hill function, as described by Toyoshiba et al. (2004), using the following formula:


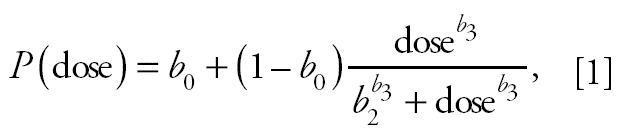


where *P*(dose) is the probability that an animal will have a tumor, *b*_0_ is the background incidence rate (E_0_), *b*_2_ is the half-maximal dose (ED_50_), and *b*_3_ is the shape parameter. Parameters were estimated using maximum likelihood techniques assuming a binomial distribution for the tumor counts and their standard errors estimated using sandwich estimators (Zhang et al. 2000). The relative potency factor (RPF) of each congener (cong) is calculated as *b*_2,TCDD_/*b*_2,cong_. In the full model (the “independent” model), equation 1 is fit to each congener and to the mixture, resulting in separate estimates of *b*_0_, *b*_2_, and *b*_3_ for each of the three congeners and for the mixture. For the mixture, the dose is an additive function of the component congener doses and the ratios of their ED_50_ values to that of TCDD (Toyoshiba et al. 2004). With this formula for the mixture dose, if the congeners are dose additive in the mixture, then the RPF for the mixture will be 1. That is, *b*_2,Mix_ = *b*_2,TCDD_ if the congeners are dose additive.

Chi-square–based likelihood ratio tests were used to evaluate all hypotheses. The hypotheses tested were as follows:

Same shape:


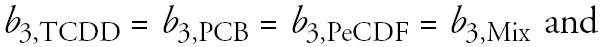


and


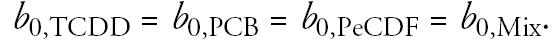


Additivity: same-shape hypothesis and


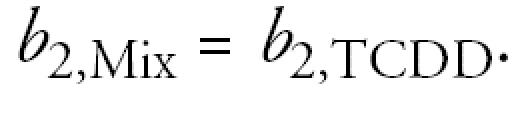


WHO: same-shape and additivity hypotheses, and


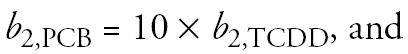


and


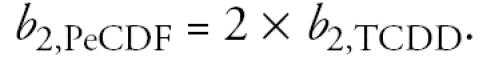


The statistical power of the likelihood ratio tests was investigated by simulating data using the maximum-likelihood estimates for the less restricted model (e.g., additivity) to evaluate our ability to reject the more restricted model (e.g., WHO TEFs). The power was found to be rather small, ranging from 0.1 to 0.5 for these data and this design.

## Results

In all four studies, there were significant increases in the incidence of both neoplastic and nonneoplastic effects in several tissues [all data from these studies are available from the NTP website (NTP 2004)]. Four specific neoplasms were observed in all of the studies, and increases in the incidences of these neoplasms were considered to be related to treatment: cholangiocarcinoma and hepatocellular adenoma of the liver, cystic keratinizing epithelioma (CKE) of the lung, and gingival squamous cell carcinoma (SCC) of the oral mucosa (Table 1). In the studies of TCDD, PCB-126, and the TEF mixture, the incidences of these neoplasms were significantly and dose-dependently increased over controls. In the study of PeCDF, the incidence of cholangiocarcinoma and hepatocellular adenoma showed a significant dose–response trend over the dose range used, whereas the incidences of CKE and gingival SCC were not significantly elevated above controls. Neither CKE nor cholangiocarcinoma was observed in control animals from any of these studies.

**Figure 1:**
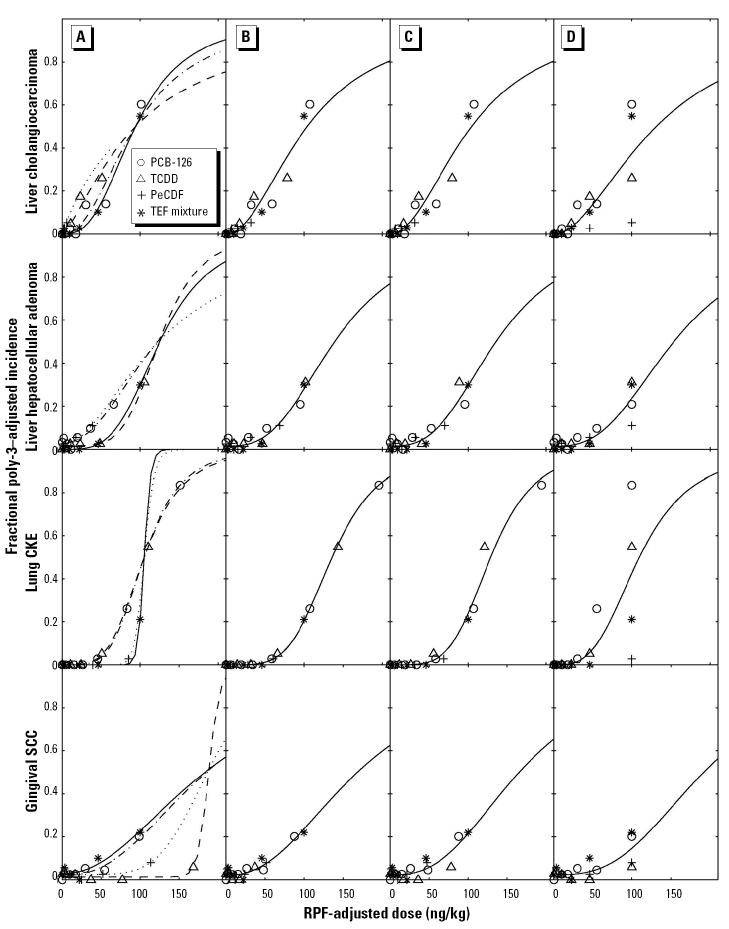
Dose–response modeling of fractional poly-3–adjusted tumor incidence showing each data set under four different model conditions: (*A*) independent model, (*B*) same-shape model, (*C*) additivity model, and (*D*) WHO model. Individual dose–response data from each respective study are shown.

The incidences of these neoplasms were used for the dose–response analysis of the several hypotheses related to the TEF approach. We tested three hypotheses in this study. First, we tested whether the shapes of the dose–response curves were the same across all four studies for each neoplasm, because this is a fundamental assumption in the TEF approach (Van den Berg et al. 1998). To achieve this, survival-adjusted (Bailer and Portier 1988) incidence data from the four studies were modeled using sigmoidal Hill functions, and differences in model fits were evaluated by maximum likelihood methods (Toyoshiba et al. 2004). Initially, each data set was modeled with parameters describing the dose response unrestricted, allowing an independent optimal fit for each chemical or mixture (Figure 1A). This model was then compared with a model in which the only parameter that was unique to each compound was the ED_50_ (Figure 1B). By comparing the error associated with the two model fits, we tested the null hypothesis that a common shape model was as good a fit as the optimal independent fit. This appeared to hold true (Table 2), indicating that each neoplasm had a common dose–response shape across all four studies. The shape of the dose–response curve for each neoplasm was highly nonlinear (shape parameter > 1.5). RPFs for each neoplasm were calculated based on the ratio of the ED_50_ (e.g., RPF PCB-126 = ED_50_ TCDD/ED_50_ PCB-126) (Table 2). In general, the RPFs for PCB-126 were similar to the WHO TEF value of 0.1 (0.11, 0.09, and 0.09 for cholangiocarcinoma, hepatocellular adenoma, and gingival SCC, respectively), except for the induction of CKE, where the RPF for PCB-126 was 0.2.

The second hypothesis to be tested was whether the increased incidence for the mixture for each neoplasm was consistent with a potency-adjusted dose-additive combination of the individual effects of TCDD, PCB-126, and PeCDF. This was achieved by comparison of the additive model to the same-shape model, for each respective neoplasm (Table 2; Figure 1C vs. Figure 1B). In both models, the administered “dose” of the mixture (on a TEQ basis) was calculated by summing the optimized RPF-adjusted dose of TCDD, PCB-126, and PeCDF


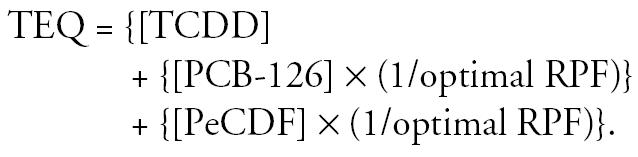


However, the ED_50_ for the additivity model uses the ED_50_ value optimized for the TCDD data set, whereas in the same-shape model the ED_50_ is optimized to the mixture data set. If dose additivity were true, then the fit of the dose–response curve for the mixture should be statistically the same as for TCDD.

For both liver neoplasms, the additive models could not be rejected (Table 2, *p* > 0.05) and showed minimal deviation from dose additivity. The optimal relative potencies for cholangiocarcinoma and hepatocellular adenoma were 0.98 and 1.02, respectively, compared with the expected value of 1.0. For CKE of the lung, there was a 1.2-fold increase over the expected value of 1.0 (Table 2), although this was not significantly different at the *p* < 0.01 level. Similarly, for gingival SCC the mixture showed only 47% of the response predicted under dose additivity (antagonism), but this was not significant at the *p* < 0.01 level.

Finally, we tested the hypothesis that the current WHO TEFs for PCB-126 (0.1) and PeCDF (0.5) (Van den Berg et al. 1998) could be used rather than the optimal RPFs. For this test, we compared the fits for the WHO model and the additive model (Figure 1D vs. Figure 1C). For the WHO model, the ED_50_ for the mixture was forced to be the same as that of TCDD, and the RPFs for PCB-126 and PeCDF were fixed as 0.1 and 0.5 rather than being optimized. In this case, the models for cholangiocarcinoma and CKE were rejected (Table 2, WHO model; *p* < 0.001). In contrast, the models for hepatocellular adenoma and gingival SCC were not rejected (Table 2, WHO model; *p* > 0.05), indicating that the dose response for the mixture was consistent with a TEF-adjusted dose-additive combination of individual congener effects. For cholangiocarcinoma, it is likely that the lower than predicted potency of PeCDF for this neoplasm (0.16 compared with the WHO TEF of 0.5) was driving this deviation. Similarly, for CKE, the higher potency of PCB-126 and lower predicted potency of PeCDF resulted in this rejection.

## Discussion

The main objective of the present study was to test the hypothesis that the increased tumor incidence observed with a mixture of dioxins could be predicted based upon the potency-adjusted dose-additive effect of the individual compounds present within the defined mixture. A key assumption in this approach is that, across the different studies, the shape of the dose–response curves for the increased incidence of each respective neoplasm is fundamentally the same. This was indeed the case, indicating that it is appropriate to describe the relative carcinogenicity of each compound/mixture by the ratio of their ED_50_ values to that of TCDD. Furthermore, we showed that the observations seen for the mixture for each of the four neoplasms were, in general, consistent with the potency-adjusted dose-additive effects seen individually for TCDD, PCB-126, and PeCDF. Finally, we showed that for hepatocellular adenoma and gingival SCC, the effects seen for the mixture were generally consistent with the use of the WHO TEF values (Van den Berg et al. 1998) of 0.1 and 0.5 for PCB-126 and PeCDF, respectively. Moreover, although the use of the WHO TEFs for increased incidences of cholangiocarcinoma of the liver and CKE of the lung was statistically rejected, the estimated potency of the mixture relative to TCDD alone for these sites was 0.98 and 1.21, respectively, only marginally different from the expected value of 1.0.

It is important to note that the current WHO TEFs are based on an expert evaluation of individual studies that examined the relative potency of a given chemical to the reference compound, TCDD, which is assigned a potency of 1 (Van den Berg et al. 1998). TEF values are an order of magnitude estimate of the overall “toxic potency” of a given compound and therefore do not specifically refer to the potency from any single study with a particular end point. By comparison, an RPF is determined for a specific chemical in a single study relative to a specific end point. Consequently, it was expected that the estimated potencies would not be identical to the WHO TEF values. It is noteworthy that although RPF values for other end points used for the derivation of TEFs span several orders of magnitude, in general the RPFs for each compound across different sites varied less than half an order of magnitude.

From these analyses, it is evident that the current WHO TEF value of 0.1 for PCB-126 is an appropriate value. For cholangiocarcinoma, hepatocellular adenoma, and gingival SCC, the optimal potencies of PCB-126 were 0.11, 0.09, and 0.09, respectively. The optimal potency for induction of CKE was 0.20. The increased potency for the mixture appeared to be due to a higher than predicted observed potency of PCB-126 for this site (0.21 compared with its WHO TEF of 0.1). Although use of an overprediction of potency would ultimately be protective of human health when used in a risk assessment setting, an underprediction of risk would be less protective. Additional research is required to understand the pathogenesis of these squamous neoplasms and if this may be related to human lung cancer risk.

For PeCDF, it appears that the current WHO TEF of 0.5 (Van den Berg et al. 1998) somewhat overestimates its potency for all the analyzed neoplasms. For cholangiocarcinoma, hepatocellular adenoma, CKE, and gingival SCC, the optimal potencies of PeCDF were 0.16, 0.34, 0.30, and 0.26, respectively. This suggests that the current TEF value for PeCDF ought to be reevaluated for its application in quantitative cancer risk assessments. The lower potency of PeCDF observed here is consistent with earlier work on the promotion of altered hepatocellular foci in rat liver in a two-stage initiation–promotion model of hepatocarcinogenesis (Waern et al. 1991). In that study, the authors estimated that the potency of PeCDF relative to TCDD was approximately 0.1, when based on weekly administered dose after an initial loading dose.

Although it is beyond the scope of this article to fully compare the present TCDD study with previously reported studies of dioxins and PCB mixtures, in general the site specificity of effects from these studies was consistent with those prior studies with TCDD and related compounds. In the feed study of TCDD conducted by Dow Chemical Company, Kociba et al. (1978) observed increased incidences of neoplasms in the liver, lung, and hard palate. The increased incidence of cholangiocarcinoma that was seen in the present series of studies has not been seen before in cancer bioassays of DLCs or PCB mixtures. A detailed comparison of study design issue and comparative dose–response modeling of data from these studies will be reported separately.

Although the focus of the data reported here was evaluation of carcinogenicity, we have previously reported on the examination of induction of cytochrome P450 data from animals killed at interim time points during the conduct of these studies (Toyoshiba et al. 2004). In that analysis, we found that in general there was lack of support for common dose–response shape for induction of CYP1A1 and CYP1A2 in the liver and CYP1A1 activity in the lung. Moreover, when modeling the data under the assumption of common shape, there was in general a lack of dose additivity for the mixture. This appeared to be driven for the most part by the dose response for induction of P450 by PeCDF, which showed higher levels of induction of P450 than the other compounds. PeCDF can sequester in the liver at high levels, leading to high body burdens of this compound at higher doses. Given that the induction of these P450s is tightly linked to tissue levels of the compound, the variation in dose response likely reflects differences in short-term pharmacokinetics and pharmacodynamics. In contrast, neoplasia in these studies appears to be a more protracted response that requires longer durations of constant exposure. Hence, pharmacokinetic and pharmacodynamic differences between the compounds may not be as influential on the ultimate dose–response models.

The data presented here support additivity for compounds whose primary mechanism of action is via the AhR. However, it was not designed to address additivity for compounds that may have multiple modes of action that are also included in the current TEF scheme, for example, mono-*ortho*-PCBs (e.g., PCB-118). In addition, because exposure to PCBs always occurs as a mixture, this study was not designed to address whether the potency of a DLC is affected by non-DLCs such as the di-*ortho*-PCBs (e.g., PCB-153). To this end, additional studies as part of this evaluation of the dioxin TEF scheme being conducted by the NTP are examining the carcinogenicity of PCB-153 and PCB-118 and also mixtures of PCBs (PCB-126 and PCB-153, and PCB-126 and PCB-118).

In summary, to our knowledge this is the first study that has systematically examined the specific interactions within a mixture of compounds in the context of the chronic rodent carcinogenicity bioassay. The main conclusion from this study is that we cannot reject the hypothesis of potency-adjusted dose additivity for induction of rodent neoplasms for a defined mixture of DLCs. Moreover, the optimal potency of the defined mixture was almost the same as for TCDD alone. These analyses underscore that the use of TEFs and dose additivity for assessing mixtures of persistent AhR ligands is reasonable for cancer risk assessments and is now supported by some experimental evidence.

## Figures and Tables

**Table 1:** Summary of survival-adjusted neoplasm incidences.

Study, dose (ng/kg)*^a^*	Cholangiocarcinoma	Hepatocellular adenoma	CKE	Gingival SCC
TCDD (TEF = 1.0)				
0	0^b^**	0**	0**	2.5**
3	0	0	0	5.7
10	0	0	0	2.6
22	2.9	0	0	0
46	10.3	2.6	0	10.2
100	54.9**	29.9**	21.1**	22.0**
PCB-126 (TEF = 0.1)				
0	0**	3.2**	0**	0**
30	0	5.2	0	2.6
100	2.5	2.5	0	2.5
175	0	0	0	2.7
300	13.6*	5.5	2.7	5.4
550	14.0*	9.7	26.0**	4.7
1,000	60.3**	20.9*	83.5**	20.2**
PeCDF (TEF = 0.5)				
0	0*	2.4**	0	2.4
6	0	0	0	5.2
20	0	2.7	0	2.7
44	2.6	0	0	0
92	2.8	5.5	0	2.8
200	5.4	10.9	2.7	8.1
TEF mixture*^c^*				
0	0**	0**	0**	2.7
10	0	2.5	0	2.5
22	4.8	2.4	0	0
46	17.4	2.5	5.1	0
100	26.0**	31.0**	54.7**	6.0

a Animals were treated with each compound or a mixture with each respective dose, 5 days/week for up to 104 weeks (*n* = 53–55/group).

b All table values represent the poly-3–adjusted neoplasm incidence (%) after adjustment for intercurrent mortality.

c Mixture of TCDD, PCB-126, and PeCDF (ng TEQ/kg).

*p < 0.05 and

**p < 0.01 [in the 0-dose rows, *p*-values are for the poly-3 trend test (Bailer and Portier 1988); for other doses, these *p*-values represent pairwise comparisons between the individual dose groups and the control group].

**Table 2:** Dose–response parameter estimates of models.

	Independent*^a^*						
	TCDD	PCB-126	PeCDF	TEF mixture	Same shape*^b^*	Additivity*^c^*	WHO*^d^*
Cholangiocarcinoma							
*E*_0_ (%)	0	0	0	0	0	0	0
Shape	2.81 (0.68)	2.23 (0.58)	1.02 (1.1)	1.40 (0.43)	2.02 (0.31)	2.02 (0.3)	1.9
ED_50_ (ng/kg)	94 (9.0)	928 (112)	3,006 (9,686)	128 (32)	104 (13)	104 (10)	131
RPF, PCB-126		0.10 (0.02)			0.11 (0.02)	0.11 (0.02)	0.10
RPF, PeCDF			0.03 (0.10)		0.16 (0.04)	0.16 (0.04)	0.50
RPF, TEF mixture				0.74 (0.20)	0.98 (0.16)	1.0	1.0
*p*-Value*^e^*					0.40	0.90	< 10^−4^
Hepatocellular adenoma							
*E*_0_ (%)	0	0.03	0.01	0.02	0.02	0.01	0.01
Shape	3.74 (1.5)	2.24 (1.5)	1.86 (1.9)	4.90 (0.8)	2.95 (0.64)	2.91 (0.7)	2.80
ED_50_ (ng/kg)	125 (18)	1,896 (1,007)	645 (838)	81 (5)	141 (21)	137 (18)	155
RPF, PCB-126		0.07 (0.04)			0.09 (0.02)	0.10 (0.01)	0.10
RPF, PeCDF			0.19 (0.25)		0.34 (0.08)	0.35 (0.07)	0.50
RPF, TEF mixture				1.54 (0.24)	1.02 (0.18)	1.0	1.0
*p*-Value					0.17	0.32	0.19
CKE							
*E*_0_ (%)	0	0	0	0	0	0	0
Shape	23.4 (—)*^f^*	4.45 (—)*^f^*	16.96 (—)*^f^*	4.16 (—)*^f^*	4.45 (0.8)	4.57 (0.88)	3.61
ED_50_ (ng/kg)	121 (—)*^f^*	695 (—)*^f^*	333 (—)*^f^*	110 (—)*^f^*	136 (14)	129 (10)	109
RPF, PCB-126		0.17 (—)*^f^*			0.20 (0.02)	0.19 (0.02)	0.10
RPF, PeCDF			0.36 (—)*^f^*		0.30 (0.08)	0.34 (0.05)	0.50
RPF, TEF mixture				1.27 (—)*^f^*	1.21 (0.14)	1.0	1.0
*p*-Value					0.99	0.033	< 10^−4^
Gingival SCC							
*E*_0_ (%)	0.03	0.02	0.03	0.01	0.02	0.02	0.02
Shape	2.14 (—)*^f^*	2.42 (—)*^f^*	5.54 (—)*^f^*	26.6 (—)*^f^*	2.35 (1.0)	2.72 (1.0)	2.90
ED_50_ (ng/kg)	188 (—)*^f^*	1,905 (—)*^f^*	331 (—)*^f^*	116 (—)*^f^*	171 (51)	168 (38)	195
RPF, PCB-126		0.10 (—)*^f^*			0.09 (0.02)	0.09 (0.02)	0.10
RPF, PeCDF			0.57 (—)*^f^*		0.26 (0.12)	0.24 (0.14)	0.50
RPF, TEF mixture				1.62 (—)*^f^*	0.467 (0.25)	1.0	1.0
*p*-Value					0.93	0.047	0.07

SEs of parameter estimates are shown in in parentheses.

a Each curve had independent parameter estimates.

b The whole data set was modeled under the assumption that there is a common *E*_0_ and shape parameter across all four studies.

c The whole data set was modeled under the assumption that there is a common *E*_0_ and shape parameter across all four studies and that the ED_50_ for the mixture is based on dose additivity of the constituents (such that the RPF for the mixture is 1.0).

d The data were modeled assuming additivity and that the relative potencies for PCB-126 and PeCDF were equivalent to the WHO TEFs.

e Likelihood ratio test (analysis of the same-shape model was relative to the independent model; analysis of the additivity model was relative to the same-shape model; analysis of the WHO model was relative to the additivity model).

f Reliable SEs could not be calculated due to instability of the model.

## References

[b1-ehp0113-000043] Ahlborg UG, Brouwer A, Fingerhut MA, Jacobson JL, Jacobson SW, Kennedy SW (1992). Impact of polychlorinated dibenzo-*p*-dioxins, dibenzofurans, and biphenyls on human and environmental health, with special emphasis on application of the toxic equivalency factor concept. Eur J Pharmacol.

[b2-ehp0113-000043] Bailer AJ, Portier CJ (1988). Effects of treatment-induced mortality and tumor-induced mortality on tests for carcinogenicity in small samples. Biometrics.

[b3-ehp0113-000043] Birnbaum LS, DeVito MJ (1995). Use of toxic equivalency factors for risk assessment for dioxins and related compounds. Toxicology.

[b4-ehp0113-000043] DeVito MJ, Diliberto JJ, Ross DG, Menache MG, Birnbaum LS (1997). Dose-response relationships for polyhalogenated dioxins and dibenzofurans following subchronic treatment in mice. I. CYP1A1 and CYP1A2 enzyme activity in liver, lung, and skin. Toxicol Appl Pharmacol.

[b5-ehp0113-000043] Food and Drug Administration 2002. Good Laboratory Practice for Nonclinical Laboratory Studies. 21CFR58.

[b6-ehp0113-000043] Hamm JT, Chen CY, Birnbaum LS (2003). A mixture of dioxins, furans, and non-*ortho* PCBs based upon consensus toxic equivalency factors produces dioxin-like reproductive effects. Toxicol Sci.

[b7-ehp0113-000043] Hites RA, Foran JA, Carpenter DO, Hamilton MC, Knuth BA, Schwager SJ (2004). Global assessment of organic contaminants in farmed salmon. Science.

[b8-ehp0113-000043] Institute of Laboratory Animal Resources 1996. Guide for the Care and Use of Laboratory Animals. 7th ed. Washington, DC:National Academy Press.

[b9-ehp0113-000043] Kociba RJ, Keyes DG, Beyer JE, Carreon RM, Wade CE, Dittenber DA (1978). Results of a two-year chronic toxicity and oncogenicity study of 2,3,7,8-tetrachlorodibenzo-*p*-dioxin in rats. Toxicol Appl Pharmacol.

[b10-ehp0113-000043] NTP 2004. National Toxicology Program Homepage. Available: http://ntp.niehs.nih.gov [accessed 22 November 2004].

[b11-ehp0113-000043] Safe S (1990). Polychlorinated biphenyls (PCBs), dibenzo-*p*-dioxins (PCDDs), dibenzofurans (PCDFs), and related compounds: environmental and mechanistic considerations which support the development of toxic equivalency factors (TEFs). Crit Rev Toxicol.

[b12-ehp0113-000043] Schmidt JV, Bradfield CA (1996). Ah receptor signaling pathways. Annu Rev Cell Dev Biol.

[b13-ehp0113-000043] Stellman JM, Stellman SD, Christian R, Weber T, Tomasallo C (2003). The extent and patterns of usage of Agent Orange and other herbicides in Vietnam. Nature.

[b14-ehp0113-000043] Toyoshiba H, Walker NJ, Bailer AJ, Portier CJ (2004). Evaluation of toxic equivalency factors for induction of cytochromes P450 CYP1A1 and CYP1A2 enzyme activity by dioxin-like compounds. Toxicol Appl Pharmacol.

[b15-ehp0113-000043] Van den Berg M, Birnbaum L, Bosveld ATC, Brunstrom B, Cook P, Feeley M (1998). Toxic equivalency factors (TEFs) for PCBs, PCDDs, PCDFs for humans and wildlife. Environ Health Perspect.

[b16-ehp0113-000043] Waern F, Flodstrom S, Busk L, Kronevi T, Nordgren I, Ahlborg UG (1991). Relative liver tumour promoting activity and toxicity of some polychlorinated dibenzo-p-dioxin- and dibenzofuran-congeners in female Sprague-Dawley rats. Pharmacol Toxicol.

[b17-ehp0113-000043] Zhang J Peddada S Rogol A 2000. Estimation of parameters in nonlinear regression models. In: Statistics for the 21st Century, Methodologies for Applications of the Future (Rao CR, Szekely GJ, eds). New York:Marcel Dekker, 459–483.

